# Role of IFN-gamma and IL-6 in a protective immune response to *Yersinia enterocolitica *in mice

**DOI:** 10.1186/1471-2180-8-153

**Published:** 2008-09-19

**Authors:** Gianluca Matteoli, Edda Fahl, Philipp Warnke, Steffen Müller, Michael Bonin, Ingo B Autenrieth, Erwin Bohn

**Affiliations:** 1 Institut für Medizinische Mikrobiologie und Hygiene, Universitätsklinikum Tübingen, Tübingen, Germany; 2 IZKF Microarray Facility Medizinische Genetik, Universitätsklinikum Tübingen, Tübingen, Germany

## Abstract

**Background:**

*Yersinia *outer protein (Yop) H is a secreted virulence factor of *Yersinia enterocolitica *(Ye), which inhibits phagocytosis of Ye and contributes to the virulence of Ye in mice. The aim of this study was to address whether and how YopH affects the innate immune response to Ye in mice.

**Results:**

For this purpose, mice were infected with wild type Ye (pYV^+^) or a YopH-deficient Ye mutant strain (Δ*yopH*). CD11b^+ ^cells were isolated from the infected spleen and subjected to gene expression analysis using microarrays. Despite the attenuation of Δ*yopH in vivo*, by variation of infection doses we were able to achieve conditions that allow comparison of gene expression in pYV^+ ^and Δ*yopH *infection, using either comparable infection courses or splenic bacterial burden. Gene expression analysis provided evidence that expression levels of several immune response genes, including IFN-γ and IL-6, are high after pYV^+ ^infection but low after sublethal Δ*yopH *infection. In line with these findings, infection of IFN-γR^-/- ^and IL-6^-/- ^mice with pYV^+ ^or Δ*yopH *revealed that these cytokines are not necessarily required for control of Δ*yopH*, but are essential for defense against infection with the more virulent pYV^+^. Consistently, IFN-γ pretreatment of bone marrow derived macrophages (BMDM) strongly enhanced their ability in killing intracellular Ye bacteria.

**Conclusion:**

In conclusion, this data suggests that IFN-γ-mediated effector mechanisms can partially compensate virulence exerted by YopH. These results shed new light on the protective role of IFN-γ in Ye wild type infections.

## Background

*Yersinia enterocolitica *(Ye) is an enteropathogenic bacterium which causes gastrointestinal disorders such as, enteritis, enterocolitis and extraintestinal manifestations such as lymphadenitis, reactive arthritis, erythema nodosum, uveitis and septicaemia [1,2]. Pathogenic Ye strains carry a 70-kb plasmid (pYV), which is essential for the pathogenicity and encodes a type III secretion system (TTSS), *Yersinia *outer proteins (Yops) and YadA [3]. The TTSS enables extracellularly located yersiniae to translocate at least six effector Yops directly into host cells [3]. The Yops interfere with different signaling pathways involved in the regulation of the actin cytoskeleton, phagocytosis, apoptosis and in the inflammatory response, thus favoring survival of the bacteria in an extracellular tissue compartment [3]. YopH, YopE, YopT, and YopO/YpkA disturb cytoskeletal dynamics and thereby inhibit phagocytosis by polymorphonuclear leukocytes and macrophages [4-7]. YopH, a tyrosine phosphatase, causes disruption of focal adhesion complex structures and inhibits the oxidative burst [7,8]. Among others, the host cell targets of YopH in epithelial cells are the focal adhesion proteins Crk-associated substrate (p130Cas) and focal adhesion kinase (FAK) and in macrophages p130Cas, Fyn-binding protein (Fyb) and SKAP-HOM [8-12]. YopH mutants, which are unable to bind p130Cas, do not localize to focal complex structures in infected cells [13]. These YopH mutants showed reduced virulence in mice, suggesting that binding to p130Cas and/or Fyb is biologically relevant in *Yersinia *infections. Other functions of YopH include the inhibition of the phosphatidyl-inositol 3 kinase (PI3K)/Akt signaling pathway which is activated in macrophages upon interaction with *Yersinia *[14], and downstream effects such as expression of the chemokine monocyte chemoattractant protein-1 (MCP-1, CCL2), an important chemotactic factor for macrophages [14].

The phosphatase activity of YopH inhibits the ability of T cells to produce cytokines and proliferate, as well as the ability of B cells to express the B-cell co-stimulatory receptor CD86, possibly by dephosphorylating critical tyrosine residues on signaling proteins involved in T- and B-cell activation [14-17]. The biological relevance of YopH is underlined by different reports demonstrating that the lack of YopH results in reduced virulence of *Yersinia *in mice [18-21].

In this study, we addressed whether and how YopH might affect the innate immune response in mice. Upon infection with *Y. enterocolitica *O8 strain WA-314 (pYV^+^) or a YopH deletion mutant (Δ*yopH*) alterations in gene expression in CD11b^+ ^cells was analyzed. These cells include mostly granulocytes and macrophages and to a lower extent dendritic cells and NK cells. CD11b^+ ^cells were chosen because they are the most important spleen cell subpopulations involved in innate immune responses. The data reported herein provide strong evidence that IFN-γ and IL-6 are not necessarily required for clearance of *Y. enterocolitica *Δ*yopH *and that IFN-γ compensates YopH-mediated immune evasion mechanisms in macrophages.

## Results

### YopH deletion attenuates *Yersinia enterocolitica *in mice

**Figure 1:**
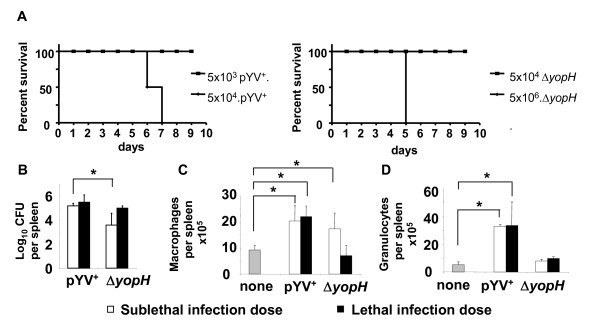
**Infection of mice with pYV^+ ^and Δ*yopH***. (A) Survival curve of C57BL/6 mice upon i.v. infection with 5 × 10^3 ^or 5 × 10^4 ^pYV^+^, or with 5 × 10^4 ^or 5 × 10^6 ^Δ*yopH *(B) The bacterial numbers in the spleen of mice and total number of (C) macrophages and (D) granulocytes in the spleen was determined 24 h after infection. The data were from two independent the means ± standard deviation of at least ten mice per group. Asterisks indicate significant differences (*p *< 0.05, A and B, logrank test; C, Wilcoxon rank test, D and E, One-Way ANOVA with Dunnett test) between the compared groups.

In this study, we addressed how the virulence factor YopH affects the early innate immune responses to *Yersinia enterocolitica in vivo*. For this purpose, we defined the inocula leading to sublethal and lethal infection after intravenous infection with pYV^+ ^and Δ*yopH*. Infection of C57BL/6 mice with 5 × 10^3 ^pYV^+ ^or 5 × 10^4 ^Δ*yopH *caused a sublethal infection, while 5 × 10^4 ^pYV^+ ^or 5 × 10^6 ^Δ*yopH *resulted in a lethal course of infection (Figure 1A). Sublethal infection with pYV^+ ^resulted in significantly higher bacterial counts in the spleen at day 1 p.i. compared with Δ*yopH*, while lethal infection with pYV^+ ^or Δ*yopH *resulted in comparable bacterial splenic counts (Figure 1B).

To address whether the sublethal and lethal infection with pYV^+ ^and Δ*yopH *affect the cells involved in innate immune responses, the number of macrophages (CD11b^+^F4/80^+^), granulocytes (CD11b^+^Ly6G^+^) and dendritic cells (CD11b^+^CD11c^+^) in the spleen was determined at day 1 p.i. The total number of macrophages was significantly increased one day after the sublethal infection with both pYV^+ ^and Δ*yopH *compared with mock-infected mice. In contrast, under lethal conditions a significant increase in the number of macrophages was only detected after infection with pYV^+ ^(Fig. 1C). The number of granulocytes was significantly increased after sublethal as well as lethal infection with pYV^+ ^but not after sublethal or lethal Δ*yopH *infection (Fig. 1D). The number of dendritic cells was similar in all conditions one day after infection (data not shown).

From this data we can conclude that by variation of the inoculum for pYV^+ ^and Δ*yopH *infections, conditions can be achieved that lead either to comparable infection course (lethal or sublethal) or to comparable bacterial burden in the spleen as well as to comparable alterations in the composition of splenic cell populations. These changes have to be considered when gene expression patterns are compared in pYV^+ ^and Δ*yopH *infections.

### Gene expression in CD11b^+ ^cells after *Yersinia *infection

To analyse whether YopH affects gene expression in cells involved in the innate immune response, splenic CD11b^+ ^cells were purified by MACS to 96–98% from mice one day p.i. RNA was extracted and subjected to gene expression analysis using Affymetrix MG-U74Av2 microarrays. First, we identified all genes which were more than 3-fold higher or more than 3-fold lower expressed after infection with either a sublethal or a lethal dose of pYV^+ ^or Δ*yopH* compared to uninfected mice resulting in 1428 probe sets. These probe sets were used in the following analyses.

### Gene expression analyses reveal differences between pYV^+ ^and Δ*yopH *infection

**Figure 2:**
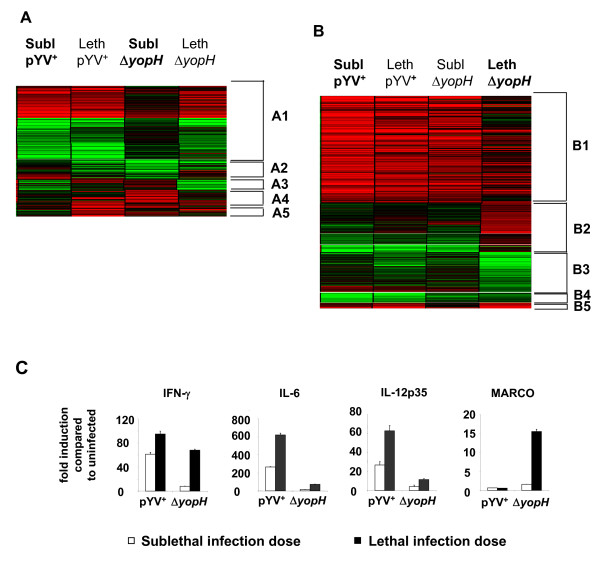
**Comparison of gene expression between mice infected with pYV^+ ^and Δ*yopH***. Microarray analysis of CD11b^+ ^cells isolated from spleen of mice infected with pYV^+ ^or Δ*yopH *for one day. All probe sets are displayed whose SLR ratio between infection with pYV^+ ^and Δ*yopH *for one day was > 1.49 or < -1.49. Heat maps indicate increased (red) or decreased (green) expression of genes in CD11b^+ ^cells one day after infection of mice compared to CD11b^+ ^cells from uninfected mice. The heatmaps indicate genes differentially expressed and clustered in different groups: (A, approach I) sublethal pYV^+ ^infection versus sublethal Δ*yopH *infection; (B, approach II) sublethal pYV^+ ^versus lethal Δ*yopH *infection. (C) CD11b^+ ^cells were enriched from uninfected mice and from mice infected for 1 day with pYV^+ ^or Δ*yopH*, and RNA was extracted. mRNA expression of IFN-γ, IL-6, IL-12p35 and MARCO was analysed and normalized to RPL8 mRNA expression. The data represent the fold induction of mRNA expression compared to CD11b^+ ^cells from uninfected mice. Representative data for three independent experiments are shown.

Two approaches were selected, to help identify immune response genes whose expression differs after infection with pYV^+ ^and Δ*yopH*. In the first approach, gene expression of CD11b^+ ^cells from mice after sublethal pYV^+ ^and sublethal Δ*yopH *infection was compared. This method was used to compare gene expression associated with similar outcome of disease; however, the different bacterial splenic counts and different composition of CD11b^+ ^subpopulations has to be considered in subsequent comparative analyses. In a second approach, we compared gene expression of CD11b^+ ^cells from mice with comparable bacterial splenic counts after infection with pYV^+ ^and Δ*yopH*. To accomplish this, we compared mice after sublethal pYV^+ ^and lethal Δ*yopH *infection resulting in similar splenic bacterial counts one day p.i. (~10^5 ^CFU) in the spleen. In both approaches k-means cluster analysis was carried out to characterize groups of co-expressed genes. Genes which were more than 3-fold differentially expressed between pYV^+ ^and Δ*yopH *infection are listed in Additional file 1 and 2 and depicted as heat maps in Figure 2.

The immune response genes identified by approach I (sublethal pYV^+ ^infection versus sublethal Δ*yopH *infection) comprised genes which were more highly expressed after both sublethal pYV^+ ^and sublethal Δ*yopH *infection, as compared with mock-infected mice. However, the expression level of these genes was more than 3-fold higher after sublethal pYV^+ ^infection (Table 1, column 1) as compared with sublethal Δ*yopH *infection (Table 1, column 3). These genes were designated as Group 1 and included genes encoding e.g. IFN-γ, IL-6, histidine decarboxylase and iNOS (Table 1, Figure 2A, Cluster A1 and Additional file 1).

**Table 1:** Immune response genes differentially expressed after infection with pYV^+ ^compared to Δ*yopH*

	**pYV^+ ^(5 × 10^3^)**	**pYV^+ ^(5 × 10^4^)**	**Δ*yopH *(5 × 10^4^)**	**Δ*yopH *(5 × 10^6^)**
	**fold expression compared to uninfected**	**fold expression compared to uninfected**	**fold expression compared to uninfected**	**fold expression compared to uninfected**
***Group 1***				
**A**				
CXCL2	39.4	45.3	7.5	36.8
CCL2	13	22.6	4.6	48.5
Histidine decarboxylase	3.5	3.8	1.2	4
IFN-γ	12.1	36.8	3.0	13.3
IL-12A	21.1	36.8	1.2	8.6
TNF-α	9.9	10.6	4	10.6
**B**				
IL6	19.7	32.0	9.2	5.2
INOS	4.6	3.7	1.3	1.7
IL1A	90.5	119.4	14.9	26.0
CXCL1	12.1	21	3	7
				
***Group 2***				
**A**				
SAA3	1.1	1.3	0.4	3.5
Lactotransferrin	0.4	0.5	0.3	2.8
LBP	0.8	1	0.5	2.8
MARCO	0.6	0.4	1.7	8.6
Orosomucoid	0.4	0.7	0.7	3.3
Peptidoglycan recognition protein	0.6	0.9	0.8	2.6
**B**				
CCL17	2.1	10.6	1.2	6.5
IL10	1.2	4	2	8.6
				
***Group 3***				
CXCL9	21.1	14.9	9.2	3.7
IIGP1	32	39.4	24.3	8.6
MX1	6.5	6.5	4	1.2
WARS	4.6	3.7	4.6	1.9
VEGFA	6.5	6.5	5.7	2.3

As the bacterial splenic load after sublethal pYV^+ ^infection was lower than that after sublethal Δ*yopH *infection at day 1 p.i., we cannot exclude the possibility that the different gene expression is due to the different bacterial load in the spleen.

Therefore, in approach II we first compared expression of Group 1 genes in mice after sublethal pYV^+ ^(Table 1, column 1) and lethal Δ*yopH *infection (Table 1, column 4) which resulted in similar splenic bacterial counts 1 day p.i. The data revealed Group 1A, which included genes with comparable expression after sublethal pYV^+ ^and lethal Δ*yopH *infection such as, IFN-γ and histidine decarboxylase, and Group 1B, which included genes which were expressed less after lethal Δ*yopH *infection as compared with sublethal pYV^+ ^infection.

Approach II also revealed genes which were more highly expressed after lethal Δ*yopH *infection compared to sublethal pYV^+ ^infection (Table 1, Group 2, Figure 2B, Cluster B2). While the expression of several of these genes (Group 2A) which include acute phase reactants such as serum amyloid alpha 3 (SAA3), lipopolysaccharide binding protein (LBP) and other genes such as MARCO, peptidoglycan recognition protein and lactotransferrin were increased only after lethal infection with Δ*yopH *compared to uninfected mice, others (Group 2B), e.g., IL-10 and CCL17 increased only after lethal infection either with pYV^+ ^or Δ*yopH *as compared to uninfected mice.

Furthermore, approach II revealed genes (interferon induced genes such as interferon inducible GTPase 1 (IIGP1), CXCL9, as well as VEGF-α) which were highly induced after *Yersinia *infection but which were lower expressed after lethal Δ*yopH *infection compared to sublethal or lethal pYV^+ ^infection.

To ensure that the results obtained by microarray analyses can be reproduced by other methods, mRNA expression analyses for IFN-γ, IL-6, IL-12p35 and MARCO were performed by Real-time RT PCR. The results obtained were comparable to those obtained by microarraray analyses (Figure 2C).

### IFN-γ and IL-6 are not necessarily required for clearance of *Y. enterocolitica *Δ*yopH*

**Figure 3:**
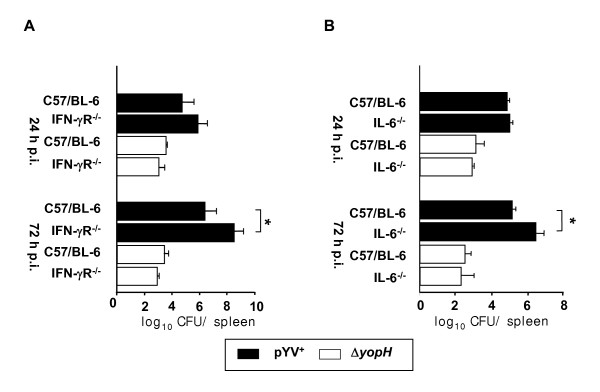
**IFN-γR and IL-6 are not necessarily required for the control of Ye Δ*yopH *infection**. IFN-γR^-/- ^(A) or IL-6^-/- ^(B) mice as well as C57BL/6 mice were infected intravenously with 5 × 10^4 ^pYV^+ ^(black bars) or 5 × 10^4 ^Δ*yopH *(white bars). The bacterial number was assessed in spleens 24 and 72 hours after infection. Values represent the average log_10 _CFU per spleen with the standard errors of the means indicated by error bars (5–10 mice per group). Asterisks indicate significant differences (*p *< 0.05, Wilcoxon Rank test) between the compared groups. Two further independent experiments showed comparable results.

The data presented above revealed that in self-limiting infections (sublethal), the expression of both IFN-γ and IL-6 in CD11b^+ ^cells increased significantly less in Δ*yopH *infection compared to pYV^+ ^infection, suggesting that these cytokines might be necessary for control of pYV^+ ^but not of Δ*yopH*. From earlier studies, it is known that IFN-γ and IL-6 are crucial for clearance of Ye wild type infection [22,23]. To investigate whether these cytokines are in fact dispensable for the early defense against Ye lacking the virulence factor YopH, wild type (C57BL/6), IFN-γR^-/-^, and IL-6^-/- ^mice were infected with Δ*yopH *or pYV^+ ^and the bacterial numbers in the spleen were determined. As shown in Figure 3, upon infection with pYV^+ ^the bacterial burden was higher at all investigated time points than in Δ*yopH *infected IFN-γR^-/-^, IL-6^-/- ^or wild type mice. 72 hours p.i., the bacterial burden was significantly higher (p < 0.05) in spleens of IFN-γ-R^-/- ^and IL-6^-/- ^mice compared to wild type mice after infection with pYV^+^. In contrast, a similar bacterial burden was found in IFN-γR^-/- ^and IL-6^-/- ^mice compared to wild type mice after infection with Δ*yopH*. From this data, we conclude that IFN-γ and IL-6 are not necessarily required for the control of Δ*yopH *infection at least in the early phase of infection.

### IFN-γ pretreatment compensates YopH mediated inhibition of bacterial killing by BMDM

To address whether the importance of IFN-γ, in counteracting Ye infection, could be linked to the defense against YopH mediated virulence, we investigated whether YopH may counteract the killing of yersiniae by BMDM. In addition, we tested whether IFN-γ improves the killing of yersiniae by BMDMs. For this purpose, BMDM were cultured with and without IFN-γ for 24 h and subsequently infected with either pYV^+ ^or Δ*yopH*. The number of bacteria was determined 0.25 hours after the infection of BMDM; alternatively BMDM were treated after 0.25 hours with gentamicin and the intracellular survival of bacteria was determined 3 and 5 hours after infection.

**Figure 4:**
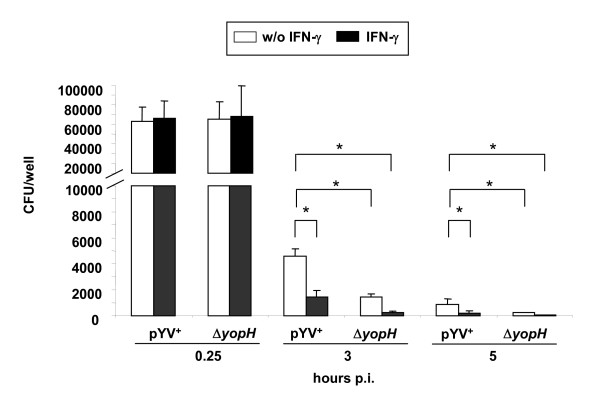
**Survival of bacteria in BMDM**. BMDM were either pretreated or not pretreated with IFN-γ (50 ng/ml) for 24 h and subsequently infected with pYV^+ ^or Δ*yopH *with a MOI of 5 (C) for 0.25 or three hours as described in Material and Methods. Numbers of bacteria were determined by a CFU assay. The data represent the CFU ± standard deviation of three independent experiments. All groups were compared for one point in time and asterisks indicate significant differences between the compared groups (*p *< 0.05, one-way ANOVA with Bonferroni correcctions).

At 0.25 hours after the infection, the number of bacteria associated with BMDM was comparable in all groups (Figure 4) suggesting that neither pretreatment of BMDM with IFN-γ nor YopH affects association of Ye with BMDM. At three hours p.i., 7 % of pYV^+ ^and 2.1% of Δ*yopH *were viable in BMDM cultured without IFN-γ indicating that killing of Δ*yopH *by BMDM cultured without IFN-γ was significantly more effective than killing of pYV^+ ^(p < 0.01). Interestingly, pretreatment of BMDM with IFN-γ significantly increased the killing of both pYV^+ ^and Δ*yopH *by more than 60 %. This data indicates that the killing of pYV^+ ^by BMDM, pretreated with IFN-γ, is as effective as the killing of Δ*yopH *by BMDM culture without IFN-γ, suggesting that IFN-γ- mediated mechanisms are not necessarily required for Δ*yopH *infection but contribute to an effective immune response to pYV^+^.

## Discussion and conclusion

YopH is one of the most important virulence factors of Ye, which promotes colonization and survival of Ye in lymphoid tissues of mice. This capability appears to be attributed to its ability to inhibit phagocytosis [5,24]. and T cell activation through the intracytoplasmic action of YopH in phagocytes and T cells, respectively [15-17].

The goal of this study was to investigate whether and how YopH might interfere with the innate immune response *in vivo*. To this end, the gene expression profiles prior to and after infection with pYV^+ ^or Δ*yopH *were analyzed in CD11b^+ ^cells from the spleen of infected mice. CD11b^+ ^cells were chosen because they represent the most important spleen cell subpopulation involved in innate immune responses such as granulocytes, macrophages, and dendritic cells and have been demonstrated to be involved in or targeted by Ye infection.

The major problem with *in vivo *studies using Ye mutants is that mutations in major pathogenicity factors such as YopH affect virulence of Ye *in vivo*. Consequently, infection of mice with the same infection dose of wild type and mutant Ye ultimately results in different bacterial burden in infected organs. This gives rise to (i) different immune stimulation by Ye compounds and, as demonstrated herein, (ii) different recruitment of inflammatory cells and hence (iii) different composition of CD11b^+ ^spleen cell subpopulations. As a result, the differences in gene expression between wild type and mutant Ye might reflect both direct and indirect effects of virulence factors such as YopH. As an alternative, mice can be infected with different doses of wild type and mutant Ye, which will lead to comparable bacterial burden in infected organs such as the spleen. In this study we used both approaches and compared gene expression in mice infected with identical doses of wild type and mutant Ye (leading to different bacterial burden and clinical outcome) and variable doses (leading to comparable bacterial burden and clinical outcome). However, in the latter approach we could not rule out effects in gene expression that may result from different spleen cell subpopulations. Additional comparisons were performed, such as comparison of sublethal wildtype with lethal wildtype infection, as well as sublethal Δ*yopH *with lethal Δ*yopH *infection because the information obtained was mostly redundant to those already received with the comparisons discussed below.

We focused on the differences in genes known to be involved in immune responses. Sublethal pYV^+ ^and lethal Δ*yopH *infection results in the same bacterial burden in the spleen but shows some differences in the composition of the cell populations expressing CD11b such as granulocytes, macrophages and dendritic cells. However, comparison of the expression between sublethal pYV^+ ^and lethal Δ*yopH *infection reveals that acute phase reactants and other genes involved in innate immune response such as the antimicrobial component lactoferrin and MARCO (playing a role in clearance of pneumococcal infections) [25] are increased after Δ*yopH *infection compared to pYV^+^infection. Since acute phase reactants are predominantly constitutively expressed, the differences in gene expression after sublethal pYV^+ ^and lethal Δ*yopH *infection may be due to changes in the composition of the cell populations which express CD11b but nevertheless may be also associated with lethal course of Δ*yopH *infection.

IL-10 and CCL17 were found to be higher expressed after sublethal pYV^+ ^versus lethal pYV^+ ^infection as well as after sublethal versus lethal Δ*yopH *infection, respectively. Since each of these conditions was associated with a comparable composition of the cell populations expressing CD11b in the spleen we conclude that increased expression of IL-10 and CCL17 is not due to differences in the composition of CD11b^+ ^cells. Previous studies suggested that the secreted Ye protein LcrV of O8 strains binds to TLR2 and triggers increased IL-10 secretion in macrophages [26]. In line with this, it was shown that infection with an inocolum of pYV^+ ^which is lethal for wildtype mice can be survived by IL-10^-/- ^mice [27] indicating that IL-10 is a marker for lethal or sublethal course of infection. However, this might not be a general observation for all *Yersinia *strains or species since TLR2 activity and probably IL-10 secretion induced by LcrV vary due to a hypervariable region in the N-terminus of LcrV [28]. In addition, the highest TLR2 activity was found for O8 strains as used in this study. Likewise, CCL17, a chemokine which attracts Th2 and regulatory T cells [29-31]. could also be involved in driving infections in direction of a lethal course; this, however, has to be more thorougly investigated in further studies.

Since a high number of genes showed a lower expression after sublethal Δ*yopH *infection we focused predominantly on this aspect. Within this group several proinflammatory response genes dependent on TLR signaling such as IL-12, CXCL1, CXCL2 and TNF-α but also IFN-γ were predominantly found. Since cytokines and chemokines act as secreted proteins the overall expression levels may be of more relevance than the subpopulation of cells which expresses these genes. In addition, histidine decarboxylase was found in this group of genes. The expression of histidine decarboxylase seems to reflect the importance of histamine signaling for control of Ye infection [32]. The improved reduction of the bacterial burden as found after infection with a sublethal dose of Δ*yopH *is associated with a lower expression of such genes. The weaker immune response found after infection with Δ*yopH *may reflect the more effective clearance of bacteria but may also raise the possibility that components of this immune response are no longer required to control the infection. Previous data suggested that IL-12, IL-18 and TNF-α may not be required for clearance of Δ*yopH *infection [19]. In line with these findings we have shown that IFN-γ and IL-6 may also not be required for clearance of a Δ*yopH *infection. Thus, clearance of infection in the spleen is comparable in C57BL/6 and congenic IFN-γR^-/- ^mice. Similar experiments were performed using IL-6^-/- ^mice, indicating that IL-6 is not necessarily required for clearance of Δ*yopH *infection.

Other groups found that oral infection of BALB/c mice with a *Y. pseudotuberculosis yopH *mutant resulted in a similar bacterial burden in the spleen five days after infection of IFN-γ^-/- ^and IFN-γ^+/+ ^mice [20,21]. Interestingly, by co-infection of *Y. pseudotuberculosis *wild type and *yopH *mutant it has been demonstrated that in the presence of IFN-γ the *yopH *mutant is more efficiently killed in comparison to wild type. On the contrary, in the absence of IFN-γ the survival advantage of the wildtype *Y. pseudotuberculosis *compared to the *yopH *mutant is much weaker [20]. This data supports the view that IFN-γ is required for clearance of wildtype *Yersinia *[22] but plays a minor role for clearance of Δ*yopH *mutant and would explain at least in this case similarities between *Y. pseudotuberculosis *and Ye infection of mice.

There is substantial knowledge about IFN-γ mediated effector mechanisms in host defense against pathogens, such as promotion of TH1 responses, induction of expression of 47 kDa GTPases [33], oxidative burst or antigen presentation [34]; however, the exact effector mechanisms induced by IFN-γ for clearance of a *Yersinia *infection are not yet defined. By determining killing of BMDM upon infection with Ye we demonstrate that killing of yersiniae by BMDM is quite efficient. However, in contrast to Δ*yopH*, pYV^+ ^is less efficiently cleared by BMDM indicating that YopH inhibits killing of yersiniae to some extent. In addition pretreatment of BMDM with IFN-γ bypasses indirectly or directly YopH mediated resistance of killing of yersiniae. This data further supports the idea that IFN-γ is not necessarily needed to defeat *Yersinia *lacking YopH. However, YopH may also exert other immune evasion mechanisms. In fact, YopH also inhibits T cell activation [15-17]. Previous data demonstrates that infection of athymic, T cell deficient C57BL/6 nude mice leads to an increased bacterial number in the spleen starting only three days after infection compared to infection with C57BL/6 mice suggesting that T cell responses are already involved in the early phase of Ye infections [35].

Taken together, the data presented herein show that IFN-γ bypasses or compensates for immune evasion mechanism of YopH. Moreover, we provide evidence that improvement in killing of bacteria by macrophages may represent part of the effector functions of IFN-γ to compensate for immune evasion mechanisms provided by the interplay of the effector Yops of the Ysc-Yop type three secretion system and that deletion of YopH enables the immune defense to work efficiently without the presence of IFN-γ. However, further studies need to define whether alterations in the immune response against YopH deletion mutant are solely due to the phosphatase activity of YopH or whether they also may be due to changes in the secretion of some of the other Yops (e.g. YopT, YopP) [36]. Moreover, future studies are needed to define the crucial mechanisms important for clearance of *Yersinia *infection *in vivo *and to elucidate which IFN-γ induced genes are important to bypass *Yersinia *mediated inhibition of yersiniae killing. However, since beside IFN-γ other genes such as IL-6, TNF-α, IL-12, IL-18 are not necessarily required for clearance of Ye infection with Δ*yopH*, it can be speculated that YopH does not counteract the effector functions of one of those genes specifically but rather a proinflammatory response in general.

## Methods

### Bacterial strains and plasmids

For infection the Ye O:8 strain WA-314 (pYV^+^) [37], and derivatives of this strain were used. The strain WA-C (pYV^-^) lacks the pYV virulence plasmid and the strain WA-C pYV *yopH *Δ17–455 (Δ*yopH*) lacks YopH [36].

### Mouse infections

The mouse strains used were C57BL/6j-IL-6^*tm1Kopf *^(referred as IL-6^-/-^) kindly provided by M. Kopf, ETH Zürich, Switzerland [38] C57BL/6J-*Ifng*^rtm1agt ^IFN-γR^-/- ^[39] purchased by Jackson laboratories and corresponding C57BL/6 wild-type mice. Six- to eight-week-old female C57BL/6 mice were infected intravenously with the indicated doses of Ye strains from frozen stock suspensions. The administered dose was determined by plating serial dilutions on Mueller-Hinton agar for 36 h at 27°C. For kinetic analysis, mice were asphyxiated using CO_2 _at various time points post infection. Spleens were aseptically removed and homogenized in 5 ml HBSS-buffer (Hank's Balance solution supplemented with 2% FCS and 10 mM HEPES). To determine the numbers of CFU/organ, serial dilutions of homogenated organs were plated on Mueller-Hinton agar plates. Infection experiments were approved according to German law by the Regierungspräsidium Tübingen (H2/02).

### Selection of splenic CD11b^+ ^cells

For the selection of splenic CD11b^+ ^cells for further use in microarray experiments five to ten mice per group were sacrificed by CO_2_asphyxiation. The spleens were removed and placed in ice-cold HBSS (Ca^2+ ^and Mg^2+ ^free Hanks' balanced salt solution; BioWhittaker, Walkersville, MD), supplemented with 2% FCS (HyClone, Logan, Utah) and 10 mM HEPES buffer. The spleens were forced with a 5 ml syringe pestle through a 100 μm-pore nylon mesh cell strainer (Falcon; BD Biosciences). Red blood cells were lysed from spleen samples by incubating the cell suspensions for 5 min at room temperature in erythrocyte lysis buffer (170 mM Tris, 160 mM NH_4_Cl, pH 7.4) followed by two washes in ice-cold HBSS. CD11b^+ ^cells from a pool of five to ten mice per group were purified by positive selection using magnetic activated cell sorting (MACS)^® ^CD11b MicroBeads (Miltenyi Biotec) according to the manufacturer's instructions. To ensure enrichment values of 96–98% purity, the magnetic separation was performed twice. Cell viability and number were assessed by trypan blue exclusion.

### Flow cytometry

Splenic single-cell suspensions were obtained as described above. 1 × 10^6^cells were resuspended in 100 μl FACS buffer and stained with surface marker-specific fluorophore-conjugated antibodies (Abs). The following Abs and secondary staining reagents conjugated with different fluorophores were used for flow cytometric studies: rat anti-mouse Ly-6G (Gr-1: RB6-8C5), rat anti-mouse CD4 (RM4-5), rat anti-mouse CD8a (53-6.7) or rat anti-mouse CD45R/B220 (RA3-6B2); rat anti-mouse CD19 (1D3), rat anti-mouse pan-NK-cell (DX5), rat anti-mouse CD3ε chain (145-2C11) and the hamster anti-mouse CD11c (HL3), rat anti-mouse CD11b (M1/70), streptavidin-FITC, streptavidin-PE; all from BD Pharmingen, Heidelberg, Germany. The rat anti-mouse F4/80 (CI:A3-1) and rat anti-mouse MARCO (ED31) from Serotec (Serotec, Oxford, UK) were also used. The specificity of the staining was verified by the use of isotype control mAbs. Samples were analyzed on a FACSCalibur flow cytometer (BD Immunocytometry Systems) with gating on the propidium iodide-negative cells.

### Isolation of total RNA and microarray analysis

Total RNA was extracted from CD11b^+ ^cells derived from a pool of five to ten mice per group using the RNAeasy mini-kit following the manufacturer's instructions (Qiagen, Hilden, Germany). Generation of fragmented cRNA was performed as recently described [40] and used for hybridization onto MG-U74Av2 from Affymetrix (Affymetrix, High Wycombe, UK). Genechips were washed, stained with streptavidin-phycoerythrin and read using a GeneChip Scanner 2500 (Affymetrix) at the IZKF microarray facility, Tübingen.

### Microarray data analysis

Analysis of microarray data was performed using the Affymetrix Microarray Suite 5.0, Affymetrix MicroDB 3.0 and Affymetrix Data Mining Tool 3.0. All array experiments were scaled to a target intensity of 150, otherwise the default values of the Microarray suite were used. Filtering of the results was performed as follows: signal log_2 _ratios (SLR) of the experiments for each colonization condition and time point was calculated. A median SLR greater than 1.5 or less than -1.5 was considered as significant change. The absolute detection calls and change calls were assigned using the detection p-values or the change p-values. A detection p-value of ≤ 0.04 was considered as present (P), a detection p-value of > 0.04 and ≤ 0.06 was considered marginal (M) and a detection p-value > 0.06 was considered absent (A). A change call of increase (I) was assigned with a median change p-value of ≤ 0.0025 and a change call of marginal increase (MI) was assigned at a median change p-value > 0.0025 to 0.003. Change calls of marginal decrease (MD) were assigned at a median change p-value of ≥ 0.997 to < 0.998 and a change call of decrease (D) was assigned at a p-value ≥ 0.998. All others were assigned no change (NC). Of the probe sets with a significant SLR that had a change call other than NC in comparison to uninfected and were not absent in both compared groups were retained. Probe sets with an increase but a detection call of A in the infected cells were also discarded. Of the remaining probe sets only those with a signal at least 3 times higher than the average noise were used for further analysis. The magnitude and direction of expression changes were estimated as Signal Log Ratio (SLR). Microarray data have been deposited in NCBIs Gene Expression Omnibus (GEO,  accession number (GSE 11189). For each condition described here one microarray was used.

### Cluster analysis

For Cluster analysis we used Genesis, release 1.6.0 (Institute for Genomics and Bioinformatics, University of Technology, Graz, ). To analyze the relationship between groups of genes we performed a k-means clustering with a number of 10 clusters and a maximum of 200 iterations. Categorization was based on the NetAffx database .

### Quantitative RT- PCR analysis

Total RNA from CD11b^+ ^cells was extracted using the RNeasy Mini Kit (Qiagen). 2 μg of RNA were reverse transcribed as described [41]. Real-time RT-PCR was carried out on a GeneAmp 5700 Sequence Detection System (Applied Biosystems, Darmstadt, Germany). Each 20-μl reaction contained 10 μl Platinum Quantitative PCR SuperMix-UDG (Invitrogen, Karlsruhe, Germany), 0.4 μl ROX Reference Dye (Invitrogen, Karlsruhe, Germany), 3.6 μl PCR grade water, 1 μl target gene specific Assay-on-Demand Gene Expression Assay Mix Mm00446190_m1 for mouse IL-6, Mm00443258_m1 for mouse TNF-α, Mm00434165_m1 for mouse IL-12 p35, Mm00440265_m1 for mouse MARCO, or Mm00657299_g1 for mouse RPL8 (including primers and dye-labeled hybridization probes; Applied Biosystems, Darmstadt, Germany), and 5 μl cDNA. Thermal cycling conditions for all reactions were as follows: 2 min at 50°C, 10 min at 95°C, then 40 cycles of 15 s at 95°C and 1 min at 60°C. Results were quantified using the 2^-ΔΔC^_T _method [42,43]. Cytokine mRNA expression levels were normalized to the expression of the houskeeping gene ribosomal protein L8 (RPL8). All PCR experiments were performed in duplicate, and standard deviations were calculated and displayed as error bars.

### Analysis of intracellular survival by CFU assay

Intracellular survival was determined with modification as previously described [44]. 2 × 10^5 ^BMDM resuspended in DMEM (Invitrogen, Karlsruhe, Germany), containing 10% FCS (Sigma, Taufkirchen, Germany), supplemented with Na-pyruvate at 1 mM (Biochrom, Berlin, Germany), and 2 mM L-glutamine (Invitrogen, Karlsruhe, Germany) were seeded into 24 well cell culture plates and incubated overnight. Bacteria were grown overnight at 27°C in LB medium and subcultivated for 2 h at 37°C. The bacteria were washed twice with PBS and finally diluted as needed in DMEM supplemented with 10% heat inactivated fetal calf serum, 2 mM L-glutamine and 1 mM Na-pyruvate. The bacterial number was diluted to a MOI of 5. After addition of bacteria cell culture plates were centrifuged for 5 minutes at 200 × g to facilitate contact of bacteria. After 15 minutes of infection cells were washed twice with PBS. Some of the wells were used to determine initial number of cell associated bacteria by a CFU assay. To the remaining cells cell culture medium containing 8 μg per ml gentamicin was added. After one hour the medium was removed and complete cell culture medium was added containing 4.5 mg/ml gentamicin. Three or five hours after infection a CFU assay was performed as described recently [44].

### Statistics

The data shown in the figures are from representative experiments. Differences between mean values were analyzed as indicated using either Wilcoxon Rank test, one way ANOVA analyses or logrank test by using Graph Pad Prism software . A *p *value of < 0.05 was considered statistically significant.

## Authors' contributions

GM, EF and PW were equal partners in the production of these findings and all three participated in the design of the study and carried out the infection experiments, ex vivo and *in vitro *experiments, performed data analyses and helped to draft the manuscript. SM guided and supported performance of the microarray analyses. MB processed the RNA for microarray experiments and performed microarray experiments. IBA participated in the study design, data analysis and draft of the manuscript. EB conceived of the study, carried out the design and coordination and drafted the manuscript.

## Supplementary Material

Additional file 1 **Comparison of sublethal pYV^+ ^versus sublethal Δ*yopH *infection**. List of genes which fulfill the criteria that signal log_2_ratio (SLR) after sublethal pYV^+ ^(infection with 5 × 10^3 ^Ye) compared to sublethal Δ*yopH *infection (infection with 5 × 10^4 ^Ye) is > 1.5 or < -1.5. Expression levels of genes after infection with sublethal or lethal pYV^+ ^as well as Δ*yopH *infection are depicted as fold induction compared to uninfected mice. Click here for file

Additional file 2 **Comparison of sublethal pYV^+ ^versus lethal Δ*yopH *infection**. List of genes which fulfill the criteria that signal log_2_ratio (SLR) after sublethal pYV^+ ^(infection with 5 × 10^3 ^Ye) compared to sublethal Δ*yopH *infection (infection with 5 × 10^6 ^Ye) is > 1.5 or < -1.5. Expression levels of genes after infection with sublethal or lethal pYV^+ ^as well as Δ*yopH *infection are depicted as fold induction compared to uninfected mice. Click here for file
